# Early Childhood-Onset Prosopometamorphopsia Following Respiratory Tract Infection With Serological Evidence of Mycoplasma pneumoniae Exposure: A Pediatric Case Report

**DOI:** 10.7759/cureus.102888

**Published:** 2026-02-03

**Authors:** Watanabe Yusuke, Shinichiro Morichi, Takeshi Kezuka, Gaku Yamanaka

**Affiliations:** 1 Department of Pediatrics and Adolescent Medicine, Tokyo Medical University, Tokyo, JPN; 2 Department of Ophthalmology, Tokyo Medical University, Tokyo, JPN

**Keywords:** alice in wonderland syndrome, clinical case report, mycoplasma infections, prosopometamorphopsia, visual perception distortion

## Abstract

We report a pediatric case of prosopometamorphopsia (PMO), a rare disorder characterized by distorted face perception. Following a respiratory tract infection at four years of age, the male patient began to perceive visual distortions in human faces. His developmental and medical histories were unremarkable. He had no neurological abnormalities, and his blood tests showed elevated serum levels of *Mycoplasma pneumoniae* antibodies (1:160). Urinalysis, developmental examination, head image testing, and electroencephalography revealed no obvious abnormalities. In his drawings of human faces, only the faces, ears, and hair regions were elongated, both vertically and horizontally. Based on the patient’s characteristic facial distortions, consistent drawings, and the exclusion of other neurological, structural, and developmental causes, we diagnosed PMO following a respiratory tract infection. To our knowledge, this is the first reported case of PMO characterized by early childhood onset and a possible post-infectious trigger. We hypothesize that PMO may have been triggered by an autoimmune or inflammatory process following exposure to *Mycoplasma pneumoniae*, given the absence of other structural causes. When a child reports these symptoms, it is important to consider that they may genuinely perceive them, and PMO should be included in the differential diagnosis rather than dismissing the report as a joke or imagination.

## Introduction

Prosopometamorphopsia (PMO) is a very rare disorder. Its primary symptom is visual distortion specific to the human face, which can negatively impact quality of life. Perceptual distortions in PMO are hypothesized to relate to an imbalance of activity across the face-selective network, which can recover over time through recalibration of network activity. In adults, PMO is most frequently associated with posterior cortical lesions, migraine, or epilepsy [1]. To our knowledge, no cases of this condition with an early childhood onset or triggered by infection have been reported in the literature. Herein, we describe the case of a Japanese male pediatric patient aged four years who developed PMO following an upper respiratory infection, possibly involving Mycoplasma pneumoniae infection.

This article was previously presented at the 8th Case Conference during the 29th Annual Meeting of the Japanese Society of Neuroinfection on October 24, 2025.

## Case presentation

**Figure 1:**
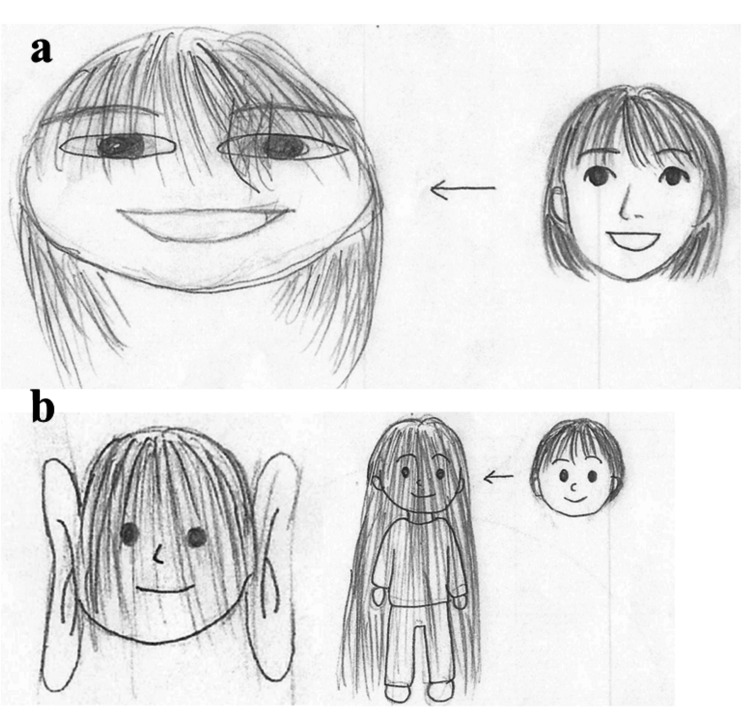
Visualization of distortion in prosopometamorphopsia. (a) A portrait drawn by the child with prosopometamorphopsia appears to be stretched horizontally relative to the original face. (b) A portrait of the patient’s brother, drawn by the mother based on the descriptions given by the patient. The ear and hair appear vertically sketched.

A four-year-old Japanese male patient was born at full term to healthy, non-consanguineous parents. At birth, he did not experience neonatal asphyxia or any other notable abnormalities. He had no significant past medical history, and there was no family history of dementia, psychiatric disorders, or neurological conditions, including epilepsy and migraine. His developmental milestones were appropriate for his age, with normal language, motor, and social development before the onset of facial perceptual distortions. A few days after recovering from an upper respiratory infection, he was brought to an ophthalmology clinic with a complaint of visual distortions of human faces, ears, and hair. Ophthalmological examinations, including fundus findings, eye movement, visual acuity (1.2 in both eyes with no astigmatism or other abnormalities), intraocular pressure (R = 13.0 mmHg, L = 12.0 mmHg), Titmus stereo test, and light reflexes testing, revealed no abnormalities. No corneal damage was observed. The patient was referred to the Department of Pediatrics and Adolescent Medicine for further examination. He showed no neurological abnormalities, and his blood tests showed normal results; however, the serum levels of M. pneumoniae antibodies were high (1:160). The patient underwent urinalysis, chest and abdominal radiography, head magnetic resonance imaging, magnetic resonance angiography, and electroencephalography, all of which revealed no obvious abnormalities. His intellectual abilities and psychological health were assessed using the Tanaka-Binet Intelligence Scale-V and Parent-Interview Autism spectrum disorder Rating Scale-Text Revision (PARS-TR) [2]. The intelligence quotient was 123, and the results of the PARS-TR indicated no tendency toward autism spectrum disorder (ASD) (three points). In the drawings of human faces that the patient brought with him, some of which were partially assisted by his mother, the facial features, ears, and hair were elongated, both vertically and horizontally (Figure 1).

Based on the specific complaint of facial distortion, the characteristic findings in his drawings, and the exclusion of other neurological, ophthalmological, and psychiatric etiologies, a diagnosis of post-infectious PMO was made. At the time of writing this report, the patient was five years of age, and on his last visit, his metamorphopsia persisted unchanged, with no progression or resolution, and his development and psychological status remained normal. The patient will continue to be followed with periodic clinical interviews, with further intelligence quotient (IQ) or psychological assessments considered as clinically indicated.

## Discussion

PMO is a rare disorder of visual perception that causes human faces to appear distorted. It constitutes one of approximately 40 types of metamorphopsia associated with *Alice in Wonderland Syndrome* (AIWS), which represents a general term for perceptual distortion [3]. The most common etiologies of PMO include brain infarction, epilepsy, hemorrhagic stroke, and surgical complications. Conversely, infection, which represents the most common etiology of AIWS, has not been reported as an etiological agent for PMO [1].

Although Epstein-Barr virus infection is the most commonly reported infectious cause of AIWS [3], cases associated with *M. pneumoniae* infection have also been documented. Omata et al. reported a case of a seven-year-old girl who developed AIWS following *M. pneumoniae* infection. She experienced the perception that her mother’s face, fingers, and other objects appeared smaller than before the onset of the infection, which we considered indicative of micropsia-type metamorphopsia [4]. The authors hypothesized that the symptom was induced by an autoimmune mechanism, which is one of the two proposed pathways through which *M. pneumoniae* can cause central nervous system involvement [5]. In the present case, the onset of symptoms was preceded by an upper respiratory tract infection. The temporal association with a respiratory illness and the elevated *M. pneumoniae* antibody titers, in the absence of any other identified etiology, suggested a post-infectious mechanism, potentially involving autoimmune-mediated disruption of the visual face-processing network. Due to the heterogeneous nature of AIWS symptoms, it is not surprising that there are differences between our case and those reported by Omata et al. [4]. As with AIWS, the definition of facial distortion in PMO is not yet fully established. For instance, while some cases involve visual distortion limited to the face, others have reported symptoms extending to adjacent areas such as the ears and the occipital region, including the hair [1]. Therefore, even when visual distortion extends beyond the face, as in the present case, the condition can still be classified as a form of PMO.

The mechanisms of face perception have been extensively studied using techniques such as functional magnetic resonance imaging and event-related potentials [6-8]. A particularly influential model in this field is Haxby's neural framework [9], which was developed based on the cognitive model proposed by Bruce and Young [10]. This framework consists of two interconnected systems: a core system that processes the invariant features of faces, and an extended system that is involved in processing changeable facial aspects such as expression and gaze. Building on this foundation, Duchaine and Yobel developed a revised model that emphasizes the modular organization of face-selective areas, with clearer functional distinctions between regions involved in identity recognition and those processing dynamic facial information [11]. This approach posits that perceptual distortions result from an imbalance in activity across this face-selective network, which is corrected by recalibration of network activity over time [11]. In a systematic review, Blom et al. reported that cases of bilateral or non-lateralized facial distortion were most often associated with lesions in the right hemisphere (61%) or bilaterally (30%) [1]. We hypothesized that the effects of an upper respiratory infection may have been associated with such lesions; however, no abnormalities were detected on neuroimaging to confirm this.

According to a review by Dirk et al., the youngest patient reported with PMO was 19 years old, and no instances of PMO triggered by infection were identified [1]. Recently, a case of PMO in a 12-year-old boy was reported, but the presumed cause in that case was epilepsy [12]. Thus, to our knowledge, there have been no previous reports of PMO occurring in early childhood, nor any instances presumed to be triggered by infection, making the present case the first of its kind. The fact that PMO can occur in children who contract relatively common infectious diseases, such as *M. pneumoniae*, suggests that PMO should be carefully considered as a differential diagnosis in children with symptoms of perceived facial distortion.

The lack of pediatric PMO reports in the literature may be related to the age of predilection for the underlying disease (e.g., brain infarction and hemorrhagic stroke) [1]. Other contributing factors include difficulty in recognizing subtle perceptual distortions in children, reliance on self-reported symptoms, which are harder to elicit in younger patients, and the lack of systematic neuropsychological or visual-perception assessments in routine clinical practice. Furthermore, the transient nature of symptoms and limited awareness of PMO among clinicians likely contribute to underreporting. Together, these challenges highlight the value of documenting pediatric cases to enhance understanding of the condition and to inform future clinical care.

The recovery time for PMO is reported to range from hours to years, with most patients achieving complete recovery; only a small minority fail to recover fully [1]. However, in a follow-up study of AIWS, symptoms persisted in 40% of patients after a mean follow-up of six and a half years [13]. The present patient’s clinical course has been followed for one year, and ongoing clinical follow-up is warranted to clarify the long-term outcome. PMO symptoms often resolve spontaneously over time; however, when an underlying treatable cause is identified, addressing it may lead to earlier symptom improvement [1]. On the other hand, PMO has been reported to induce intense fear and distress in patients due to visual distortions [14]. In one reported case involving a 12-year-old boy, such a fear significantly impaired his social functioning [12]. Therefore, it is crucial not only to conduct thorough investigations to identify and treat the underlying disease but also to provide patients with education that enables them to fully understand the causes of their symptoms, helping to cope with the condition and reduce anxiety. In this case, the respiratory infection thought to be the underlying cause had already resolved, and regular clinical follow-up did not suggest any apparent psychological impact. However, it should be noted that pediatric patients may not be able to express their complaints as clearly as adults can.

One key limitation of this report is that we inevitably evaluated the patient’s visual perception based on his drawing ability. Although the Cambridge Face Memory Test was considered, it was deemed inappropriate for this patient due to their young age and anticipated difficulty in task engagement. Recently, Antônio et al. realistically visualized the distortions experienced by a patient with PMO whose vision was distorted when he perceived human faces in person but not when they were projected on a digital screen [15]. The widespread use of such technology may allow patients with visual and sensory problems to express their symptoms clearly and, thus, allow appropriate treatments to commence sooner.

Second, additional psychological assessments, imaging assessments (e.g., functional magnetic resonance imaging), and infection-related tests beyond serum antibody titers were not performed. In addition, the mild elevation of serum *M. pneumoniae* antibody titers does not clarify whether a prior infection was causally related to the patient’s condition. Therefore, if diagnostic uncertainty remains or if new or progressive neurological symptoms emerge during follow-up, additional evaluations-including advanced neuroimaging and infection-related investigations, such as cerebrospinal fluid analysis or PCR testing-should be considered, while carefully weighing their invasiveness.

Finally, although during routine clinical follow-up to date, no overt signs of psychological distress have been observed, objective assessments specifically targeting emotional or affective symptoms were not performed. To evaluate potential underlying anxiety or distress, future cases should actively consider using age-appropriate tools, such as child anxiety scales, mood questionnaires, and behavioral checklists, to assess emotional well-being, fear, or social functioning.

## Conclusions

To our knowledge, this is the first reported case of PMO both in a child of such a young age and with infection as the presumed trigger. In this case, autoimmune or inflammatory mechanisms associated with exposure to *M. pneumoniae* may have led to an imbalance within the face-selective network, resulting in visual distortion. From a diagnostic perspective, this case highlights the importance of not only a multidisciplinary workup, including pediatric, ophthalmologic, and psychiatric evaluations, but also patient drawings as valuable objective evidence to support symptom interpretation and the diagnostic approach to PMO.

There is currently no specific treatment for PMO; however, the condition is often self-limiting, and treating an identifiable underlying cause may lead to earlier improvement in visual symptoms. On the other hand, PMO symptoms may occasionally persist for a prolonged period, as observed in this case; therefore, it is essential to share appropriate information about the condition with patients and provide reassurance. In particular, when a child reports these symptoms, clinicians should take these complaints seriously and avoid dismissing them as imagination or play. Supportive counseling and education are important to help the patient and family understand the condition and cope with persistent symptoms.
